# Evaluation of the Polymer Modified Tack Coat on Aged Concrete Pavement: An Experimental Study on Adhesion Properties

**DOI:** 10.3390/polym15132830

**Published:** 2023-06-27

**Authors:** Kyungnam Kim, Tri Ho Minh Le

**Affiliations:** 1 Department of Highway & Transportation Research, Korea Institute of Civil Engineering and Building Technology, 283 Goyangdae-Ro, Goyang-si 10223, Gyeonggi-Do, Republic of Korea; kimkyungnam@kict.re.kr; 2 Faculty of Civil Engineering, Nguyen Tat Thanh University, 300A Nguyen Tat Thanh Street, District 4, Ho Chi Minh City 70000, Vietnam

**Keywords:** tack coat, polymer modified asphalt, trackless, tensile bonding strength, shear bonding strength

## Abstract

This study addresses the challenges of overlaying old concrete pavement with asphalt by introducing a new trackless tack coat material containing polymer. The aim is to enhance the durability of asphalt concrete overlay pavement on old cement concrete pavement. It contributes to the development of improved construction techniques for pavement rehabilitation and highlights the need for reliable adhesion performance evaluation based on different spray amounts and surface conditions. Additionally, to evaluate the effect of the adhesion performance based on the spraying amount, a tensile adhesion test was conducted by applying spray amounts of 0.30, 0.45, and 0.60 l/m^2^ on different surface conditions. The basic and adhesion performances of the polymer-modified tack coat material are evaluated through direct tensile and shear bond strength tests. The test outcomes demonstrated that the newly developed polymer-modified tack coat material had considerably greater adhesion strength compared to the traditional rapid-setting products. Its adhesive strength was 1.68 times higher on concrete and 1.78 times higher on asphalt. The new trackless tack coat material exhibited an adhesion performance of 1.05 MPa in direct tensile strength at 0.45 l/m^2^, which was 1.21 times higher than the rapid-setting tack coat. Results also confirmed that the new tack coat material exhibits values 1.90 times greater than the conventional rapid-setting tack coat material in shear bond strength, respectively. By simulating the process of separation and re-adhesion of pavement layers caused, the new tack coat material shows a tensile adhesion strength of 63% of the original state, which is advantageous for securing the durability of the pavement. Overall, the newly developed polymer-modified trackless tack coat has been shown to effectively enhance the adhesion performance between pavement layers without process delay, highlighting the potential of the new tack coat material to enhance the durability of asphalt concrete overlay pavement on old cement concrete pavement.

## 1. Introduction

With the growth of the pavement construction industry, aging concrete pavement has become a concern for maintenance and repair measures [1,2]. One of the commonly used methods for daily maintenance is asphalt overlay pavement, which aims to improve the structural performance of existing pavement, as well as prevent rainwater from penetrating through cracks [3,4]. While asphalt pavement has several advantages such as easy maintenance and short construction time, there is a risk of damage to aged concrete pavement due to factors such as lack of interface adhesion, changes in volume of the lower layer, and poor compaction [5].

When applying the overlay method of installing asphalt concrete pavement on aged concrete pavement, a tack coat is used to ensure adhesion between the pavement layers [6,7]. Aged concrete pavement refers to the existing concrete pavement that has undergone a certain period of time since its original construction. Over time, the concrete pavement may experience various forms of deterioration, including aging effects caused by factors such as environmental conditions, traffic loading, and exposure to chemicals. These aging effects can lead to changes in the properties of the concrete, potentially impacting the performance and durability of subsequent asphalt overlays. Tack coat is a material that is applied to secure adhesion between the existing pavement layer or base layer and the new asphalt pavement layer on top [8,9]. It promotes integrated behavior between the layers, reducing pavement damage and extending the pavement’s life cycle [10]. When adhesion between layers is poor, the stress distribution changes [11]. Adequate adhesion ensures that the package behaves as an integrated body, and compressive and tensile stresses are evenly distributed throughout the package, ultimately increasing the lifespan of the pavement [12]. However, if the tack coat construction is poor or its performance is insufficient, adhesion between layers cannot be secured, resulting in various pavement damages such as slippage, rutting, shoving, aggregate corrugation, and potholes, becoming an indirect cause [13].

Tack coat materials such as CRS-1, a rapid-setting material, and SS-1, a slow-setting material, are commonly used in pavement construction [14,15,16]. Emulsified asphalt is another material used as a tack coat [17], which is a mixture of asphalt binder and water that can be used at room temperature [18]. However, emulsified asphalt requires a breaking time of at least 24 h for the binder and moisture to separate and the separated moisture to evaporate, which may not be feasible for construction projects with tight schedules [19]. In practice, the asphalt mixture is often laid soon after spraying the tack coat without sufficient curing time, which can lead to inadequate adhesion and pavement damage [20]. Moreover, managing the tack coat process can be challenging, as construction vehicles may cause loss of materials, and foreign substances such as fine powder and dust generated during surrounding crushing operations may impair adhesion performance [20,21]. In the case of aged concrete pavement, which has different material properties from the upper asphalt layer, higher tack coat adhesion performance is required to prevent adhesion failure and reflective cracking [22].

A number of studies have investigated the effectiveness of tack coat on aged concrete pavement. One study conducted by Apostolidis and colleagues found that the use of a polymer-modified tack coat improved the bond strength between the asphalt concrete overlay and the aged concrete pavement compared to an unmodified tack coat [8]. The study also found that the tack coat improved the resistance of the asphalt concrete overlay to reflective cracking. Another study by Sun and colleagues evaluated the performance of some different types of tack coat materials on the aged concrete pavement [23]. The study found that a polymer-modified tack coat had the highest bond strength between the asphalt concrete overlay and the concrete pavement, while an emulsified asphalt tack coat had the lowest bond strength. A more recent technique used a trackless tack coat material to improve the bond strength between the asphalt concrete overlay and the aged concrete pavement [24,25]. The study found that the trackless tack coat material improved the bond strength and reduced the risk of delamination between the asphalt concrete overlay and the concrete pavement compared to a conventional tack coat material.

Tracking refers to the problematic occurrence of bituminous materials sticking to the tires of paving equipment during the construction process when the tack coat emulsion is not applied appropriately. This can lead to detrimental consequences such as insufficient tack coat coverage in certain areas of the pavement, resulting in slippage and delamination. To address this persistent issue, a novel tack coat material called trackless tack has been developed. The trackless tack coat is specifically designed to prevent the undesired tracking or pickup of the tack coat on haul truck tires, minimizing the risk of inadequate coverage and promoting better adhesion [26]. One of the key advantages of trackless tack is its quick break time, typically ranging from 5 to 15 min. This means that the tack coat material undergoes a rapid transformation, transitioning from a wet state to a tacky state and eventually setting [27]. This quick break time allows for efficient application and reduces the chances of the material being inadvertently picked up by passing equipment. Another significant benefit of trackless tack is its ability to prevent the buildup of tack coat on haul truck tires. This characteristic is crucial as it minimizes the potential for the tracked tack coat to be transferred to other areas of the pavement, ensuring a more uniform application and reducing the risk of uneven adhesion [28]. By preventing the accumulation of tack coat on truck tires, trackless tack helps maintain the integrity of the paving process and enhances overall construction efficiency.

To further enhance the performance of tack coating for hot mix asphalt, potential approaches include exploring the temperature and aging effects on the rheological properties and performance of geopolymer-modified asphalt binder and mixtures [29], as well as investigating the rutting behavior of geopolymer and styrene butadiene styrene-modified asphalt binder [30,31]. Additionally, it is crucial to conduct a predictive analysis of how geopolymers may impact the creep recovery properties of asphalt binders, which is a critical area of investigation [32]. These research areas hold promise in improving the effectiveness and durability of tack coating for HMA applications.

Overall, these studies suggest that the use of tack coat can improve the bond strength between the asphalt concrete overlay and the aged concrete pavement, thereby reducing the risk of premature failure and costly repairs. The effectiveness of tack coat, however, is influenced by factors such as the type of tack coat material, its application rate, and the condition of the concrete pavement surface. Further research is needed to optimize the use of tack coats in pavement construction and improve the durability of asphalt concrete overlay pavements on aged concrete pavements.

However, the current research on the poor adhesion between pavement layers, inadequate tack coat adhesion performance, and reflective cracking is limited by a number of factors. One key limitation is the lack of understanding of the complex interactions that take place between different materials and layers in a pavement structure. While some progress has been made in identifying the mechanisms that lead to poor adhesion and cracking, there is still much that is not known about these phenomena. Additionally, the field is hampered by a lack of standardization in testing methods and procedures, which makes it difficult to compare results across different studies. Despite these limitations, researchers are continuing to make progress in this area, and new techniques and technologies are being developed that hold promise for improving pavement performance and durability.

Therefore, the objective of this research is to comprehensively study the factors affecting pavement interlayer bonding and tack coat performance, as well as investigate the use of innovative materials and techniques to prevent reflective cracking. The outcomes of this research will have significant implications for the design, construction, and maintenance of pavement systems, improving their performance, durability, and sustainability.

**Figure 1:**
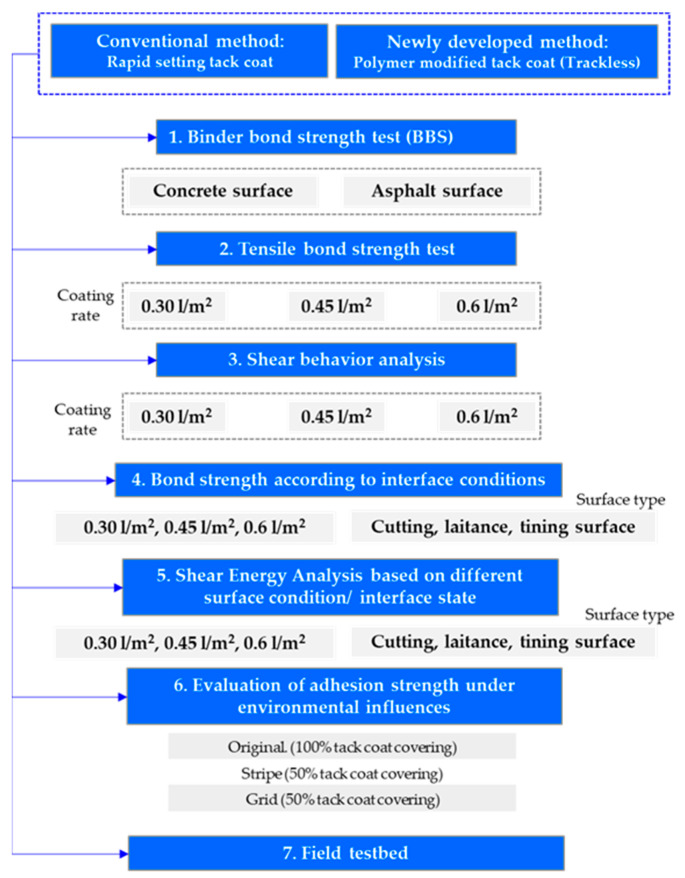
Research flowcharts.

To address the need for enhanced adhesion performance between pavement layers, a novel, and innovative trackless method has been developed, revolutionizing the traditional approach of using a tack coat. This groundbreaking method involves the simultaneous laying of an asphalt mixture without the need for a separate tack coat application. This innovative approach has the potential to significantly improve the durability of asphalt concrete overlay pavement on aged concrete surfaces. In this study, extensive laboratory testing and meticulous field evaluation were undertaken to thoroughly assess the effectiveness of a newly formulated trackless tack coat material, which incorporates a unique combination of polymers and additives. Through comprehensive direct tensile and shear bond strength tests, the fundamental and adhesion performance of the trackless tack coat material were rigorously evaluated. The findings presented in this paper highlight the pioneering nature of this novel approach and emphasize its pivotal role in advancing the field of pavement engineering. This research introduces a paradigm shift in construction practices by streamlining the process and introducing an innovative solution for achieving superior adhesion between pavement layers. Figure 1 provides a visual representation of the research methodology, summarizing the key stages of this groundbreaking study.

## 2. Materials and Methods

### 2.1. Overview of the Research Methods

#### 2.1.1. General Concept

As shown in Figure 1, this research compares the performance of two methods: the conventional method, which utilizes a rapid-setting tack coat, and the newly developed method employing a polymer-modified tack coat known as “trackless.” The study involves conducting six main tests to thoroughly evaluate the characteristics and effectiveness of these tack coats. The first test is the binder bond strength test, which is performed on both concrete and asphalt surfaces. This test aims to assess the adhesion between the tack coat and the respective surfaces. The second and third tests involve the evaluation of tensile bond strength and shear behavior. Different coating rates of 0.30 l/m^2^, 0.45 l/m^2^, and 0.6 l/m^2^ are considered in these tests to examine the influence of varying application amounts on the bond strength and shear performance of the tack coats. The fourth test focuses on bond strength under different interface conditions. Equivalent coating rates are applied on surfaces with cutting, laitance, and tinning conditions to study the impact of surface characteristics on the bonding performance. The fifth test involves analyzing the shear energy based on different surface conditions and interface states. Similar to the previous test, coating rates and surface types are varied to understand the behavior of the tack coats under different scenarios. Lastly, the sixth test evaluates the adhesion strength of the tack coats under various environmental influences. The assessment includes examining the performance of the tack coats on the original surface with 100% tack coat coverage, as well as on surfaces with stripe and grid patterns with 50% tack coat coverage. Through these comprehensive tests, the research aims to provide detailed insights into the performance and adhesion characteristics of the polymer-modified tack coat compared to the conventional rapid-setting tack coat.

**Figure 2:**
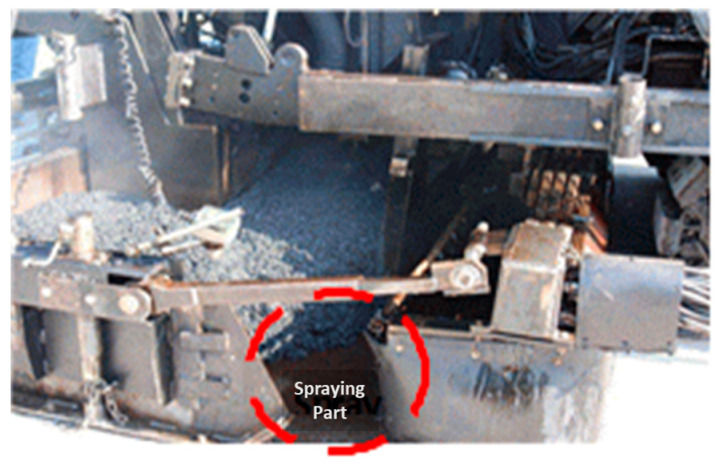
Trackless construction in South Korea.

To ensure the adhesion performance of the tack coat, improving the tack coat material’s adhesion performance and preventing damage during the construction process are crucial. One approach is to use simultaneous installation equipment, such as the Spray Paver shown in Figure 2, which attaches a tack coat spraying device to the asphalt paver and spreads the asphalt mixture while spraying the tack coat. The simultaneous laying method prevents tack coat loss caused by transport trucks and pavers by spraying the tack coat from the lower part of the paver to the back of the paver track and in front of the screed [27,28,33]. Although this method can prevent tack coat loss, it cannot secure tack coat curing time during the construction process.

Polymer modifiers are commonly used to enhance the performance of tack coat materials, particularly in the production of modified emulsified asphalt. Kim et al. (2009) found that the addition of Styrene-butadiene-styrene (SBS) and wax or Styrene-butadiene rubber (SBR) latex to emulsified asphalt can increase tensile and shear bond strengths [34]. Based on previous research, SBS, SBR, and Polyethylene (PE) have been identified as the main modifiers for modified emulsified asphalt, with SBS being effective in maintaining elasticity and viscosity at high temperatures. For this study, modified emulsified asphalt for the simultaneous laying method was produced using Lab-Plant as a modified emulsified asphalt in a rapid setting type with SBS as the modifier, taking into account the construction method of the simultaneous laying method. Quality testing showed that the modified emulsified asphalt met Texas DOT’s quality standard (CSS-1P) [35], as presented in Table 1.

#### 2.1.2. Factors Affecting Adhesion Performance of Pavement Interface

The adhesion performance of the pavement interface is affected by various factors, such as the type of emulsified asphalt used, the amount of application, and the condition of the pavement surface. In general, the adhesion performance can be improved by using a material with a high compatibility grade and securing sufficient curing time [20,36,37,38].

- Spread rate

In order to optimize adhesion performance, it is crucial to determine and apply the appropriate amount of material based on the pavement condition. The interlayer adhesion performance increases up to a certain optimum amount, beyond which a slippery film layer is formed, resulting in decreased adhesion performance [36]. Therefore, the amount of spraying should be carefully considered, taking into account factors such as pavement type, surface condition, and material properties [37,38,39,40].

- Curing time

The tack coat is composed of asphalt binder and water as an emulsifier, and it requires sufficient time for the asphalt and moisture to separate in order to achieve optimal adhesion performance. It is generally accepted that adhesion performance improves as curing time increases. Insufficient curing time can result in a significant reduction of adhesion performance, to as low as 20% of the desired level [41,42].

- Boundary state

In the maintenance section, the existing lower pavement undergoes surface pretreatment before constructing the upper pavement. Various processes such as cutting, planing, shot blasting, and water blasting are applied to aged concrete pavement depending on its type, degree of deterioration, and surface condition. To achieve optimal adhesion performance between pavement layers, the optimum amount of spraying should be applied, which changes depending on the surface texture of the lower pavement surface. Raposeiras et al. found that the surface characteristics of the lower pavement surface had a greater influence on the adhesion performance between pavement layers than the difference in tack coat performance [39]. Raab et al. reported that the interlayer adhesion performance varied depending on the treatment method of the lower pavement surface, with the cutting surface showing the greatest adhesion performance [36]. Tashman et al. stated that the loss of tack coat was reduced on the milled surface and the adhesion performance was improved [43]. Furthermore, Hwang reported that the upper and lower layer interlocking effect appeared on the tinned surface when asphalt overlay was applied, which increased the adhesion performance by increasing the tack coat application area [23].

### 2.2. Materials

#### 2.2.1. Asphalt Concrete for Overlaying of Tack Coating Purpose

For this study, asphalt concrete specimens were prepared using a mix design that was based on the Texas Department of Transportation (TxDOT) specifications [35]. The aggregate types used were crushed limestone and natural sand, with a maximum aggregate size of 19 mm. The binder type was a Performance-Graded (PG) 64-22 asphalt binder. The gradations were based on TxDOT’s Type D and E gradations, with a target air void content of 4%. The compaction process used a Superpave Gyratory Compactor (SGC) at 1.25 blows per second, and a compaction pressure of 6.9 kPa. Specimens were prepared by cutting cylindrical cores from the compacted asphalt concrete using a coring machine, with a diameter of 100 mm and a height of 63.5 mm. The cores were then saw-cut into prisms of 100 mm × 100 mm × 63.5 mm, which were used for the tack-coating tests.

**Figure 3:**
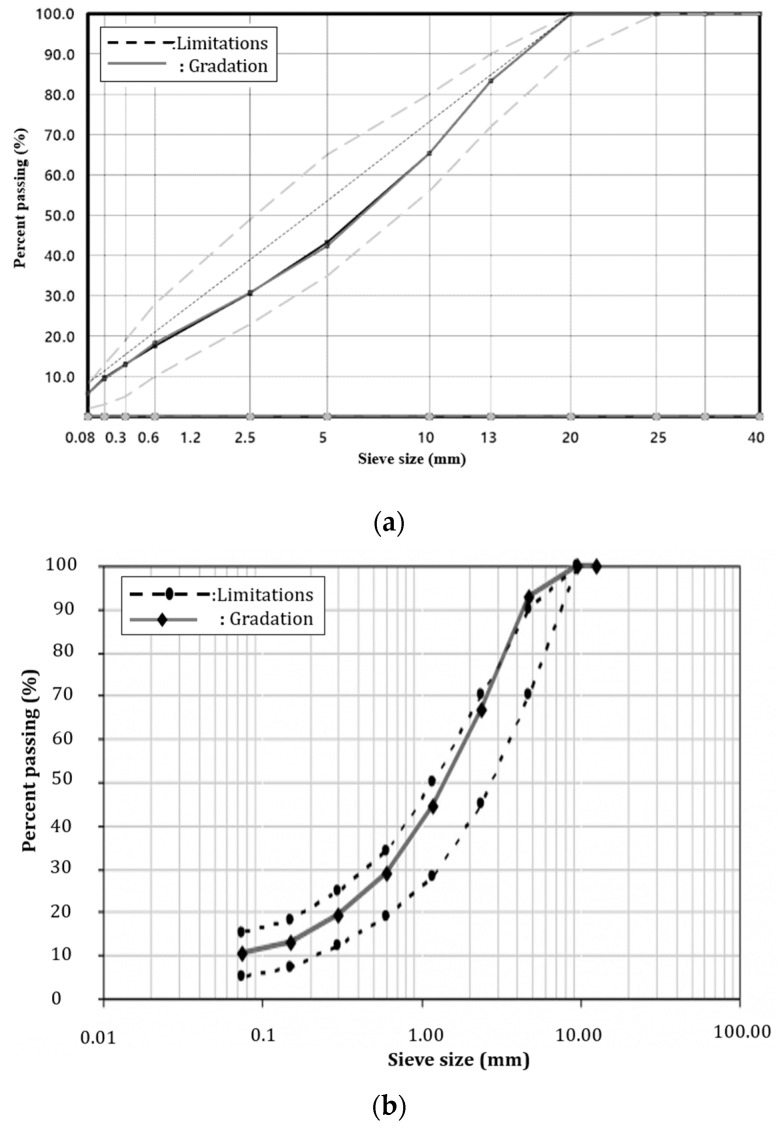
Gradation of aggregate used in this research: (**a**) in HMA mixture; (**b**) in the aged concrete mixture.

Figure 3 illustrates the aggregate gradation employed in this research, depicting two scenarios: (a) the gradation in the hot mix asphalt (HMA) mixture, and (b) the gradation in the aged concrete mixture.

#### 2.2.2. Cored Cement Concrete Samples from the Field

To investigate the performance of the tack coat on old cement concrete pavement for evaluation of adhesion strength under environmental influences, cored specimens were obtained from the existing pavement structure. The specimens were extracted using a diamond-tipped core drill, with a diameter of 150 mm and a length of approximately 300 mm. The specimens were obtained from various locations along the pavement, and care was taken to ensure that the extracted specimens were free from cracks, spalling, or any other significant damage. The cores were then cleaned using compressed air to remove any loose debris and dust and were stored in a dry location until they were ready for testing. Prior to testing, the specimens were prepared by saw-cutting the ends to obtain a flat surface and ensure that the surfaces were clean and free from any loose debris. The specimens were then coated with the tack coat material using a spray application method and were allowed to cure for a specified time period before testing. The aggregate types, cement type, and gradations of the extracted specimens were similar to those used in the development of the cement concrete specimens for the tack-coating tests.

#### 2.2.3. Tack Coating Application

To evaluate the performance of the conventional rapid-setting tack coat and the newly developed trackless tack coat on cement concrete specimens, laboratory-scale experiments were conducted. For the rapid-setting tack coat, the surface of the cement concrete specimens was cleaned and dried, and the tack coat was sprayed onto the surface at a rate of 0.3–0.6 L/m² using a conventional bituminous distributor. After a waiting period of 30 min, a new layer of hot mix asphalt was placed on the tack coat. For the trackless tack coat, the same surface preparation was conducted, and the tack coat was sprayed onto the surface at a rate of 0.3–0.6 L/m² using a special bituminous distributor with a spraying boom equipped with nozzles that do not require track creation. The spraying pattern was adjusted to provide an even coating with minimal overspray. The waiting period before the placement of the asphalt layer was less than 5 min. The application method for the trackless tack coat was designed to reduce the risk of track creation, which is a common problem with conventional tack coats that can lead to reduced adhesion and premature failure of the asphalt layer.

### 2.3. Methods for Attachment Performance Evaluation and Analysis

#### 2.3.1. Binder Bond Strength (BBS) Test

**Figure 4:**
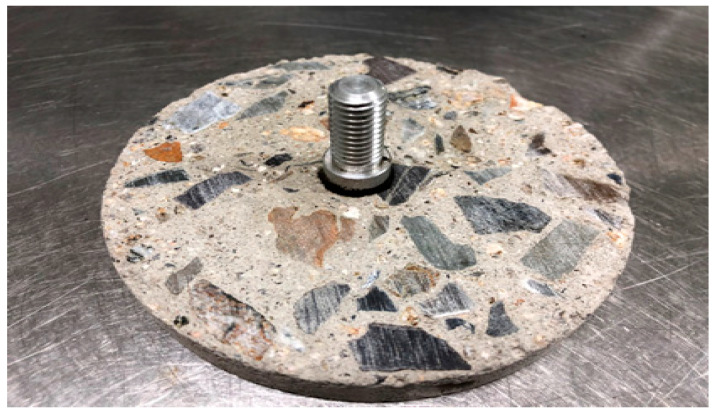
Preparation of specimens for BBS test.

The BBS test is designed to measure the adhesion between asphalt binder and aggregate and is specified in AASHTO TP-91 (Standard Method of Test for Determining Asphalt Binder Bond Strength utilizing the Binder Bond Strength (BBS) Test [44]). The experiment involves bonding the aggregate and the asphalt binder with a pull-out stub made of metal and measuring the maximum adhesion by stretching with a load of 100 psi/s. In this study, to evaluate the adhesion performance of the tack coat material between asphalt concrete and cement concrete, the BBS test was conducted as shown in Figure 4. The asphalt concrete cross-section and cement concrete cross-section were cut and a pull-out stub was attached. The experiment was conducted at 25 °C after 24 h.

#### 2.3.2. Tensile Bond Strength Test

**Figure 5:**
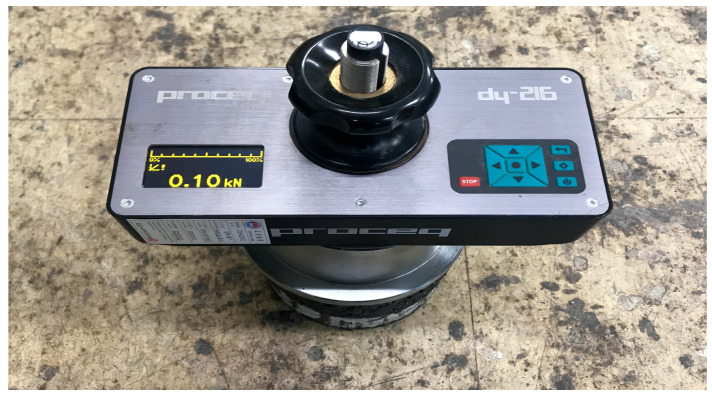
Pull-off adhesion tester.

While the BBS test is a useful method to evaluate the adhesion strength between the asphalt binder and the aggregate, it has limitations when simulating the actual field conditions where the hot asphalt mixture is laid and the tack coat moisture evaporates. Additionally, the adhesion surface of the pull-out stub is small, making it difficult to control the tack coat spraying amount. Therefore, a pull-off experiment was conducted to measure the tensile adhesion strength of the tack coat in a state similar to the actual field. A pull-off adhesion tester was used in this study as shown in Figure 5. The test was performed according to the ASTM D 4541 (Standard Test Method for Pull-Off Strength of Coatings Using Portable Adhesion Testers [45]).

**Figure 6:**
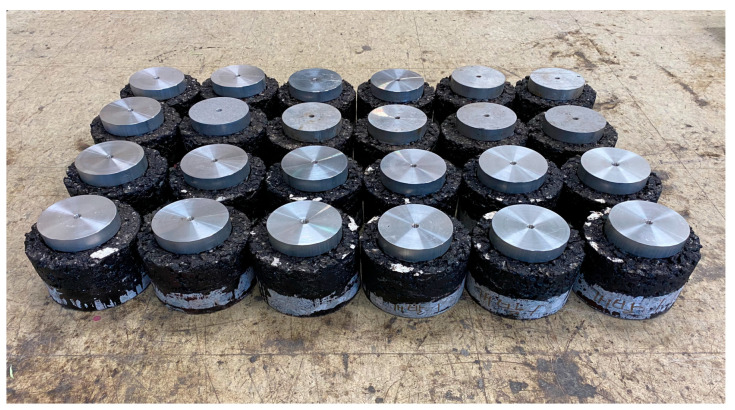
Specimens for pull-off test.

The specimen was prepared by applying the tack coat to the surface of a Φ150 mm cylindrical concrete specimen, which was cut to a thickness of 5 cm, and then immediately compacting the heated asphalt mixture on top to 5 cm using a revolving compactor. After manufacturing the specimen, as shown in Figure 6, it was cored to a depth of about 7 cm so that the adhesive surface could be separated during the tensile bond strength test.

#### 2.3.3. Shear Adhesion Strength Test

**Figure 7:**
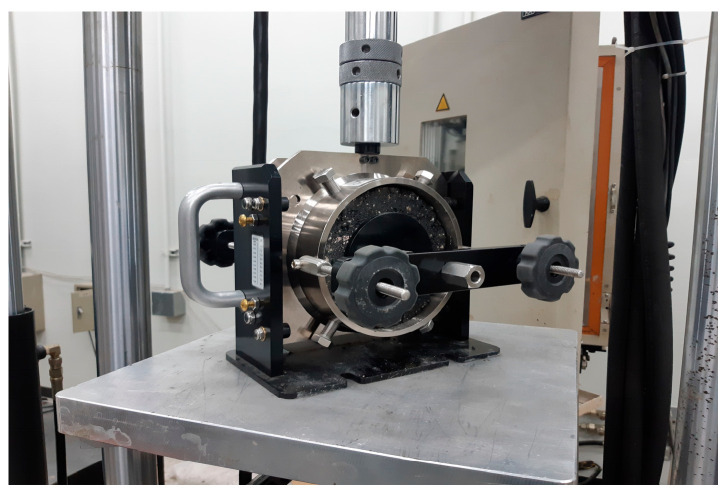
Shear bond strength test.

Adhesion between pavement layers can be formed by both chemical and physical means. In new roads, the adhesion is primarily due to the chemical bonding of the tack coat [42]. However, in cases of maintenance, physical adhesion occurs due to the road surface texture and aggregate interlocking during the existing pavement removal process. The shear adhesion strength test is a commonly used indoor test for evaluating the performance of tack coat adhesion. This test is advantageous due to its speed, high repeatability, and ability to use general laboratory equipment. The simplicity of the experiment and the standardization of the experimental procedure also makes it easy to compare and evaluate material performance. In accordance with the TEX-243-F test procedure for tack coat adhesion from TxDOT [46], the direct shear test jig was used to conduct the test as shown in Figure 7.

#### 2.3.4. Bond Strength according to Interface Conditions

Previous studies have investigated the influence of various factors on interlayer adhesion performance. Ozer et al. (2012) [37], Song et al. (2016) [47], Hou et al. (2018) [38], and Ling et al. (2019) [40] have found that interlayer adhesion is greatly influenced by the frictional force generated by the roughness of the interface mixture of composite pavement. Raposeiras et al. (2013) suggested that the characteristics of the lower pavement surface have a greater impact on interlayer adhesion performance than tack coat performance [39]. Raab et al. (2004) found that the surface treatment method of the lower pavement, such as cutting, non-cutting, and sandblasting, affects interlayer adhesion performance [36]. Tashman et al. (2008) reported that the loss of tack coat material on the milled surface tends to improve interlayer adhesion performance [43]. Hwang (2018) noted that applying an asphalt overlay to the existing concrete pavement can increase shear adhesion strength due to the interlocking effect between the concrete floor plate and the asphalt layer and the increased surface area coated with the tack coat [23]. In the case of the simultaneous laying method, adhesion performance may be affected by the use of different materials and the condition of the aged concrete pavement [48]. Therefore, in this study, the tack coat adhesion performance was evaluated by simulating the maintenance process of old concrete pavement.

**Figure 8:**
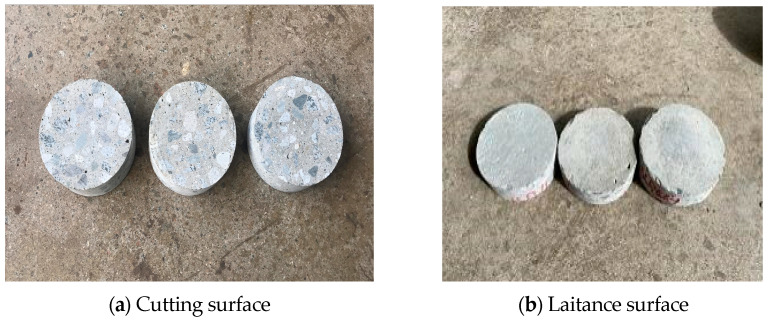

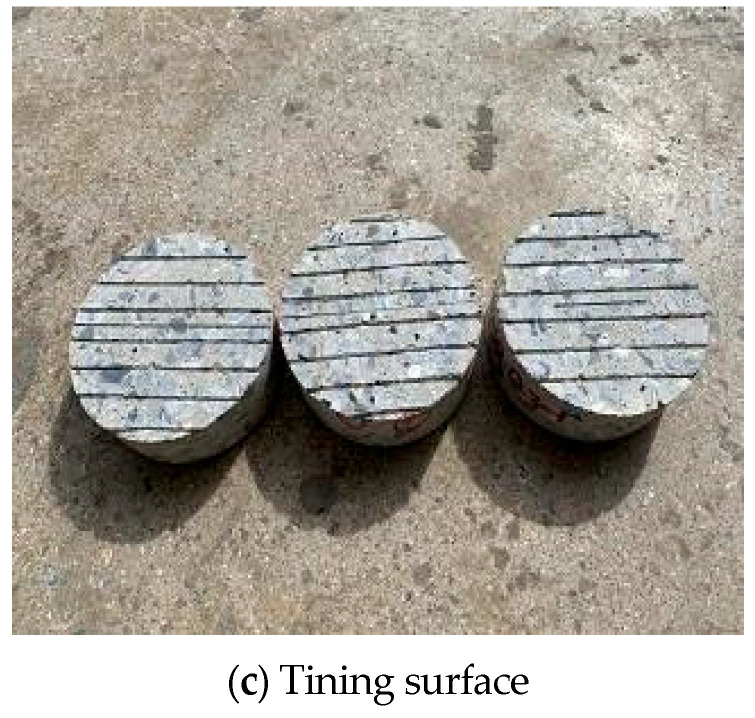
Specimens by surface condition.

First, concrete specimens were prepared for the application of concrete pavement by excluding the influence of external environmental factors, such as aggregate interlocking and foreign substance infiltration. The surface of the specimens was cleaned thoroughly to remove all foreign matter and laitance. Separate surface treatment was performed to analyze the effect of adhesion performance based on the state of the interface. The boundary conditions were divided into three categories: specimens with weathered concrete and foreign substances, specimens with laitance that were not cleaned properly during the road surface cutting process, and specimens with cut concrete or joint and tinning to prevent slipping on the road surface. The road surface tinning was installed with a gap width of 20 mm and a depth of 5 mm, as per regulations. Figure 8 shows a lower concrete pavement specimen prepared according to the interface state.

#### 2.3.5. Shear Energy Analysis Based on Surface Condition/Interface State

**Figure 9:**
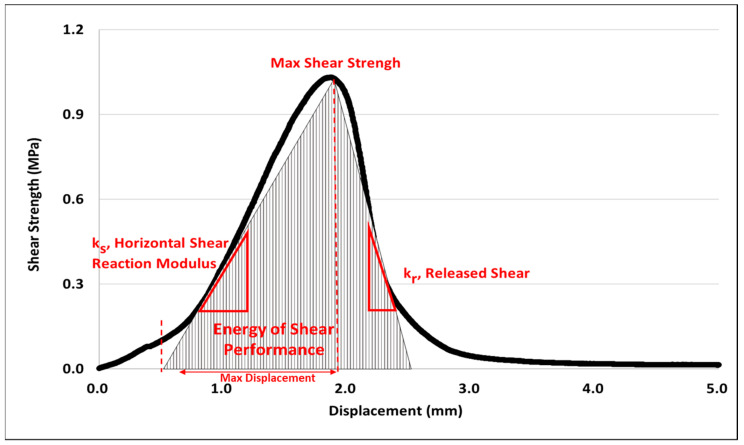
Graph of shear energy analysis.

Hwang (2018) conducted an analysis of shear energy by calculating the area under the shear strength-displacement curve [23]. Shear energy is often used as an indicator to determine pavement damage caused by interlayer bonding failure. Figure 9 shows the graph for the analysis of shear energy.

The double shear rise factor (ks) represents the slope of the maximum shear strength at the zero point of the graph, as shown in Equation (1). A higher slope, or greater shear rise factor, indicates higher stiffness of the material.

(1) $$k_{s}=\frac{\tau_{\max}}{\delta_{\max}}$$

where $\tau_{\max}$: maximum shear strength; $\delta_{\max}$: displacement at maximum shear strength.

The shear reduction factor (kr) means the slope from the maximum shear strength to the displacement that can be resisted until complete failure occurs, as shown in Equation (2).

(2) $$k_{r}=\frac{\tau_{\max}}{2(\delta_{\frac{\tau_{\max}}{2}}-\delta_{\max})}$$

where $\delta_{\frac{\tau_{\max}}{2}}$: displacement at half maximum shear strength.

#### 2.3.6. Evaluation of Adhesion Strength under Environmental Influences

During construction, materials can be lost due to the movement of construction vehicles, which can affect the adhesion performance of the tack coat [49]. In order to evaluate the effect of material loss on adhesion performance, the experiment simulated a scenario where the tack coat was partially lost. The simulation was conducted in the form of stripes and grid patterns, corresponding to about 50% of the fully coated state, to replicate the situation where the tack coat was partially lost by the wheels of the vehicle.

### 2.4. Field Application: Comparing the Effectiveness of the Conventional Tack Coating Method and the Trackless Coating Method

The purpose of this field test bed is to comprehensively compare the effectiveness of the conventional tack coating method and the trackless coating method in improving the adhesion between the old cement concrete pavement and the overlaying Warm Mix Asphalt. Situated in the Mekong Delta region of Vietnam, known for its challenging environmental conditions, this study aims to evaluate the long-term performance of both methods in real-world scenarios.

**Figure 10:**
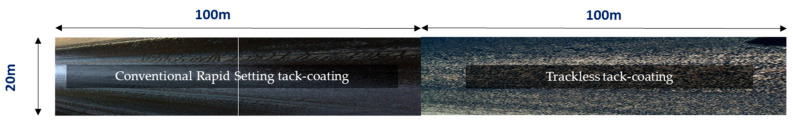
The schematic of the testbed section.

**Figure 11:**
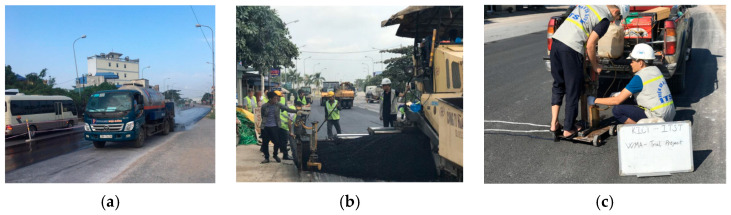
The construction of the control and testing section: (**a**) conventional rapid setting tack-coating; (**b**) trackless tack-coating; (**c**) coring of specimens after 1 year of service.

As shown in Figure 10 and Figure 11, the field testbed consists of two sections: a 100-m control section employing the conventional tack coating technique and an adjacent 100-m modified section implementing the trackless tack coating technique. The modified section utilizes a precise application rate of 0.45 l/m^2^ and applies the tining surfacing technique to enhance bonding. Both sections use the same Warm Mix Asphalt as the overlaying material, ensuring consistency in the materials employed.

To assess the long-term effectiveness of the tack coating methods, coring samples will be obtained after a service life of 1 year. These samples will be subjected to rigorous testing to evaluate the adhesion performance of the pavement under the region’s hot, humid, and rainy climate conditions. The Mekong Delta’s unique weather patterns and environmental factors pose challenges to the durability of the pavement, making it an ideal location to assess the efficacy of the two coating methods.

Furthermore, it is important to note that this field testbed focuses on a rural roadway in Vietnam, experiencing medium traffic volume. By conducting this comprehensive field test bed, valuable insights will be obtained to inform the selection of optimal tack coating methods and improve pavement construction practices, especially in regions with similar climatic conditions and traffic characteristics. The results of this study will contribute to enhancing the longevity and performance of road infrastructure in the Mekong Delta and beyond.

## 3. Results and Discussions

### 3.1. Binder Bond Strength (BBS) Test

**Figure 12:**
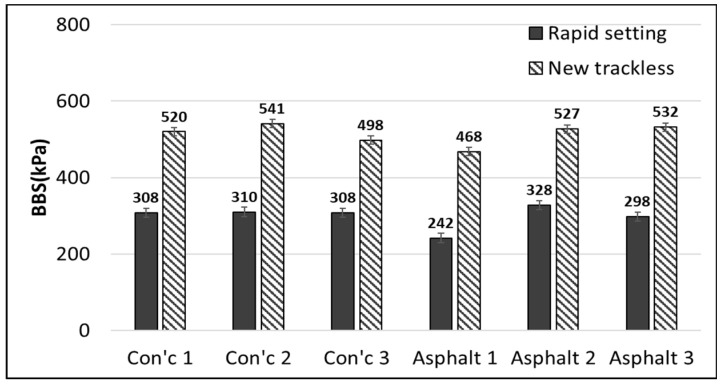
BBS test results.

In general, the newly developed tack coat material exhibited significantly higher adhesion strength compared to conventional rapid setting products, with 1.68 times higher strength on concrete and 1.78 times higher strength on asphalt, as shown in Figure 12. The experimental results show that the adhesive strength on the concrete surface was relatively higher than that of the asphalt surface. It may be due to the moisture of the emulsified asphalt being quickly absorbed into the concrete surface, facilitating moisture breaking. The results indicated that the separation and removal of binder and moisture in the tack coat material greatly affect the adhesive strength, and the concrete has an advantage in quick moisture absorption, which is favorable for tack coat curing.

**Figure 13:**
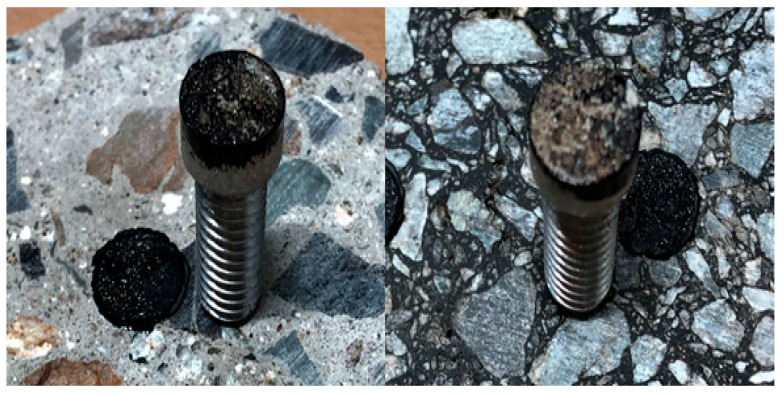
Fracture surface shape after the test.

The performance of the trackless tack coat is depicted in Figure 13, which shows that the adhesion was strong enough to pull off the underlying surface after the experiment. According to Hachiya et al. (1997) [50], the adhesion strength increases as the tack coat breaking time increases. Therefore, rapid-curing materials have an advantage in securing adhesion strength over slow-curing materials, especially in simultaneous paving where curing time is limited.

### 3.2. Tensile Bond Strength Test Results

**Figure 14:**
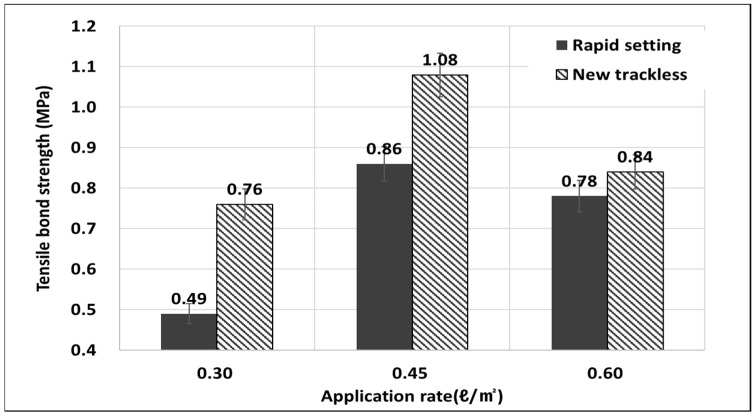
Results of tensile bond strength.

The tack coat materials used in this study were a rapid-setting tack coat and a new trackless tack coat. To evaluate the effect of the adhesion performance based on the spraying amount, a tensile adhesion test was conducted by applying spray amounts of 0.30, 0.45, and 0.60 l/m^2^. Figure 14 shows the rapid-setting tack coat and the new trackless tack coat, which are emulsions designed for simultaneous laying. The results of the tensile bond strength test at different spray amounts (0.30, 0.45, and 0.60 l/m^2^) are presented in Table 2. The highest tensile bond strength was achieved at 0.45 l/m^2^, and the strength tended to decrease when the spray amount was either larger or smaller than this value. The new trackless tack coat exhibited higher tensile adhesion strength than the rapid-setting tack coat under all spraying conditions. In particular, the new trackless tack coat material showed an adhesion performance of 1.05 MPa at 0.45 l/m^2^, which was 1.21 times higher than the rapid-setting tack coat. The addition of polymer to the emulsified asphalt generally improves adhesion performance by about 20%, and the new trackless tack coat material modified with polymer also exhibited improved performance [51].

To reduce tack coat loss during construction, a trackless tack coat is a type of emulsified asphalt that improves the properties of the applied tack coat so that it does not stick to work vehicles’ wheels. This section evaluated the tensile adhesion performance of three types of popular trackless tack coats. The results in Table 3 showed that the new trackless tack coat and the rapid setting tack coat, which are tack coat materials for simultaneous installation, demonstrated similar levels of tensile adhesion strength.

**Figure 15:**
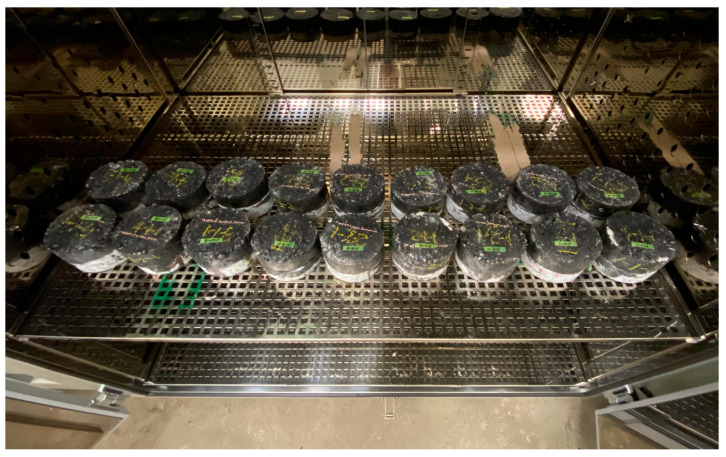
Specimen’s re-bonding in the oven.

During pavement use, the pavement layer adhered by the tack coat may separate due to deformation caused by traffic load or temperature changes. However, the primary component of the tack coat is a thermoplastic asphalt binder, allowing for reattachment even if separation occurs in the pavement layer. To simulate this scenario, the separated specimen after the tensile adhesion strength test is depicted in Figure 15. After re-bonding by its weight in an oven at 60 °C, as shown in the figure, the tensile adhesion strength was measured again.

**Figure 16:**
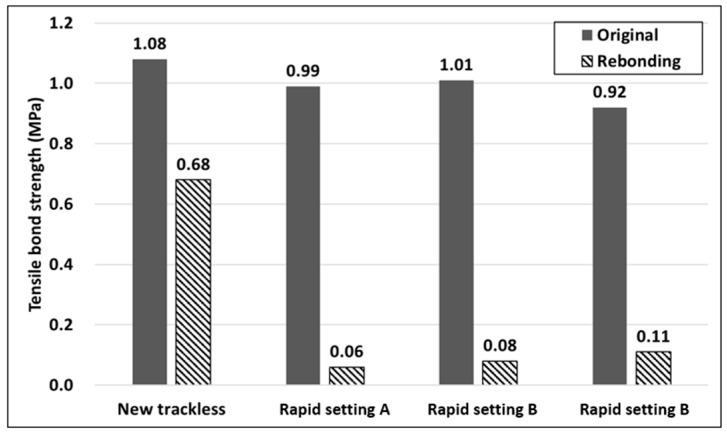
Tensile bond strength due to re-bonding.

Based on the test results presented in Table 3, it was observed that the tensile adhesion strength decreased when the previously separated adhesive surface was reattached. The trackless tack coat used in this study consists of a polymer modifier and a hard asphalt binder, which is known to exhibit a stiff behavior with less deformation upon fracturing compared to other commonly used tack coat materials [52]. Therefore, it was found to be challenging to recover the adhesive performance in case of re-attachment after separation, as illustrated in Figure 16. The re-attached rapid setting cases showed only 6.1~12.0% of the initial tensile adhesion strength, indicating that it is challenging to restore the adhesive performance once separation occurs between pavement layers. However, it was observed that the new trackless tack coat could delay pavement damage due to layer separation by exhibiting an adhesive resilience of 63% of the initial adhesive strength upon re-attachment. The unique combination of materials in the composition of this tack coat could be a factor in enhancing its adhesive properties. Additionally, the tack coat is modified with polymer, which could be a key factor in improving its performance, as polymers can enhance adhesion, durability, and other properties of asphalt materials.

### 3.3. Shear Behavior Analysis

**Figure 17:**
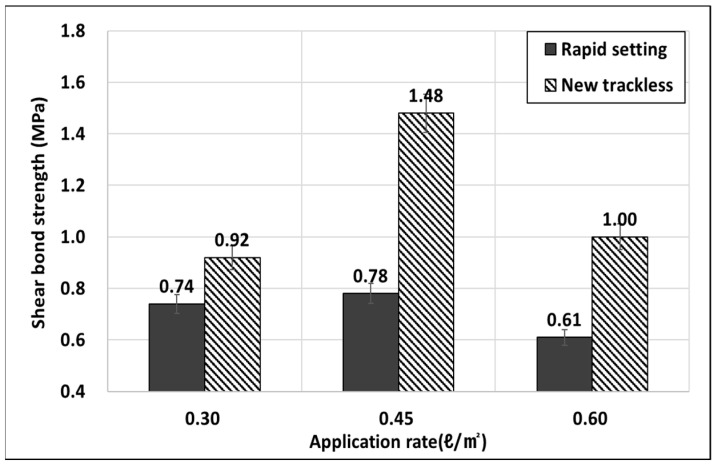
Results of shear bond strength.

The shear behavior analysis results for the adhesion test for both the rapid setting tack coat and the new trackless tack coat at different application rates are presented in Figure 17. The results show that the new trackless tack coat has a 1.90 times higher adhesion strength compared to the rapid-setting tack coat at the application rate of 0.45 l/m^2^. It is noteworthy that the adhesion strength tends to decrease at small or large application rates, similar to the trend observed in the tensile adhesion strength.

In the same manner as the tensile adhesion test, the rapid setting tack coat and the new trackless tack coat were tested at different application rates, and the results are presented in Figure 17. The experimental findings indicated that the new trackless tack coat demonstrated a value 1.90 times higher than the rapid-setting tack coat at 0.45 l/m^2^, and the strength tended to decrease when the amount of spraying was either too small or too large, similar to the tensile adhesion strength.

The United States requires a minimum shear bond strength standard of 40 to 100 psi (0.28 to 0.69 MPa) or higher [53]. Hong et al. (2017) also propose a minimum standard for tack coat shear adhesion strength of 0.6 MPa, which is similar to the US quality standard [54]. In the shear adhesion strength test conducted to simulate the simultaneous laying method, the new trackless tack coat showed a shear adhesion strength of 1.48 MPa, which was found to exhibit sufficient adhesion performance to meet the standard.

### 3.4. Bond Strength According to Interface Conditions

The experimental results showed that the optimal spraying amount of tack coat varied slightly depending on the interface conditions, as shown in Table 4. For the cutting surface conditions and conditions with laitance, which simulate the self-attachment performance of the tack coat, the optimal spraying amount was found to be 0.45 ℓ/m^2^, while for the tinning surface, it was 0.60 ℓ/m^2^. It was observed that the optimal spraying amount increased due to the increased specific surface area of the pavement to which the tack coat was applied, and the tack coat penetrated into the tinning groove, which had a certain width and depth on the tinning surface. Both the new trackless tack coat and the rapid setting tack coat met the target adhesion performance at the optimum spraying amount. However, the rapid-setting tack coat did not meet the target adhesion performance at certain spraying amounts. The new trackless tack coat exhibited 35% higher values on the cutting surface, 43% higher values on the laitance side, and 12% higher values on the tinning surface than the rapid-setting tack coat, based on the optimal spraying amount.

### 3.5. Shear Energy Analysis Based on Different Surface Conditions/Interface State

Considering the shear energy based on interface state, Table 5 indicates that the new trackless tack coat has a 36–52% lower shear reduction factor compared to the rapid-setting tack coat. It was also observed that adhesion remained intact from the point of maximum shear strength to interfacial separation. This finding suggests that the new trackless tack coat material can potentially enhance the bond strength between pavement layers, which is crucial for maintaining the durability and longevity of the pavement structure.

**Figure 18:**
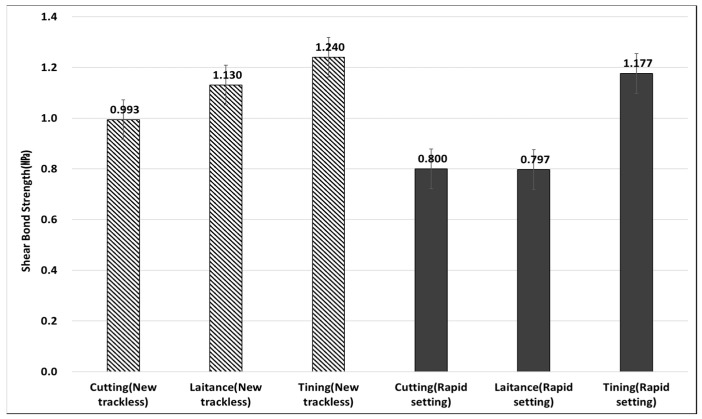
Shear Bond Strength between different testing conditions.

**Figure 19:**
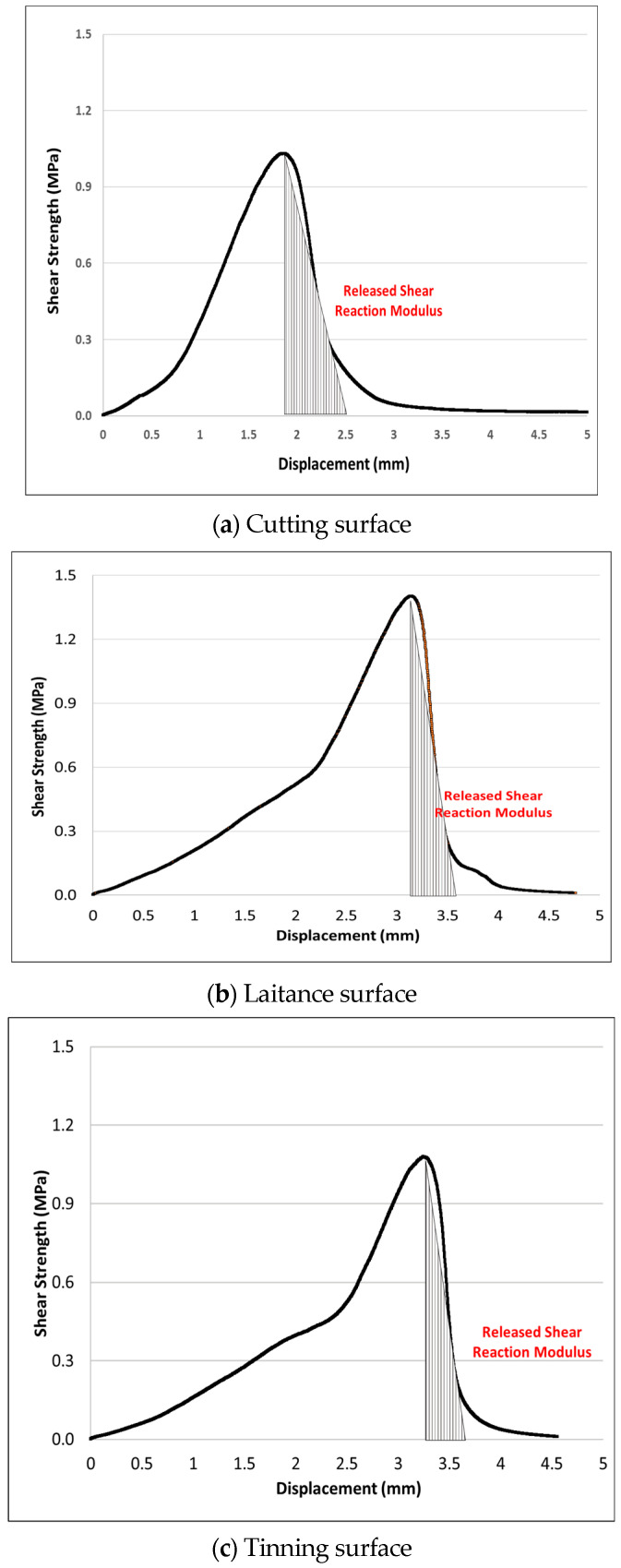
Graph of the shear test.

Additionally, the graph in Figure 18. demonstrates that the shear reduction factor for the tinning surface condition was 34% lower than that of the cutting surface, indicating that the tinning surface is more prone to separation due to a relatively small surface area where the upper and lower packages engage. Although the shear reduction factor slightly increases on surfaces where the laitance is not removed, the importance of the road pretreatment process is evident for ensuring consistent adhesion performance, as shown in Figure 19. The findings from the shear behavior analysis suggest that the surface condition of the pavement plays a crucial role in the adhesion performance of the tack coat. The tinning surface, which has a smaller surface area for engagement between the pavement layers, resulted in a significantly lower shear reduction factor compared to the cutting surface. This indicates that the tinning surface is more likely to experience separation or delamination between pavement layers. Furthermore, while the shear reduction factor slightly increases on surfaces where laitance is not removed, the results suggest that a proper road pretreatment process is critical for consistent adhesion performance. The pretreatment process ensures that the pavement surface is free from contaminants that may affect adhesion, such as dust, debris, and loose aggregate particles. These findings are important for pavement engineers and contractors to consider when selecting and applying tack coat materials. Proper surface preparation and selection of suitable tack coat materials based on the pavement condition can help to ensure optimal adhesion and enhance the durability and performance of the pavement structure.

### 3.6. Evaluation of Adhesion Strength under Environmental Influences

**Figure 20:**
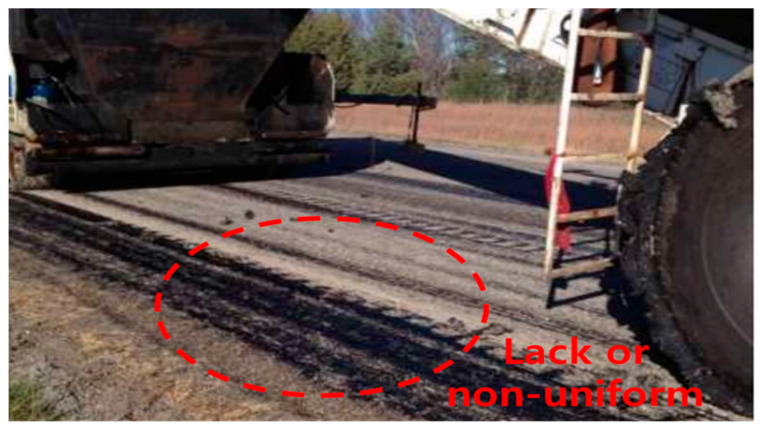
Tack coat loss by vehicle.

**Figure 21:**
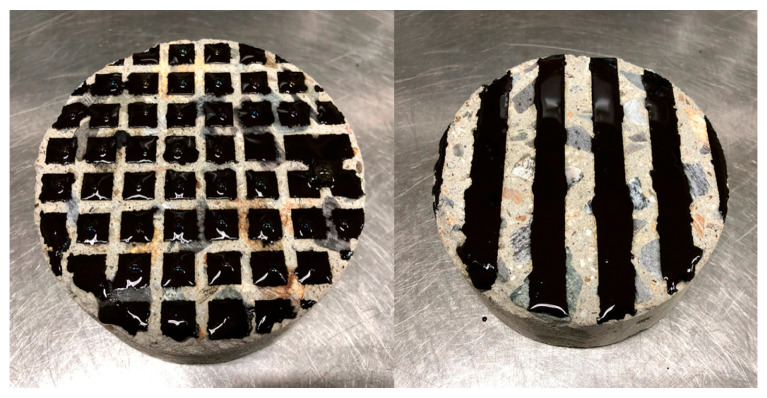
Specimens for simulating tack coat loss.

During the construction process, the tack coat can be easily lost due to the movement of construction vehicles, as illustrated in Figure 20. The loss of material can negatively impact the adhesion performance [49]. To investigate the effect of tack coat loss on adhesion, a simulation experiment was conducted as shown in Figure 21. The experiment simulated situations where the tack coat was partially lost due to vehicle wheels, creating stripe and grid patterns. The spraying amount was adjusted to approximately 50% of the fully coated state.

**Figure 22:**
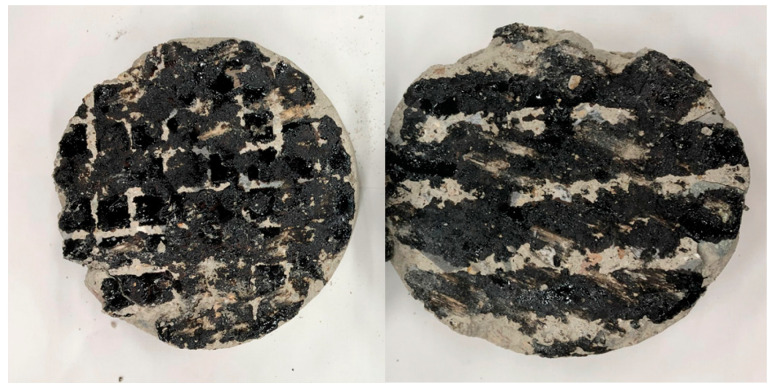
Attachment surface after the test.

The shear adhesion strength measurement results for the different levels of tack coat loss are reported in Table 6. The simulation of tack coat loss showed a decrease in shear bond strength of 11% for the stripes and 20% for the grid pattern. However, the decrease in shear bond strength was found to be smaller than anticipated, even with the reduction in the sprayed amount and 50% of the area left unsprayed, as shown in Figure 22. Surprisingly, it was discovered that the interlocking effect of the pavement layer interface contributed to the frictional resistance, which played a significant role in maintaining the shear bond strength of the pavement layers.

**Figure 23:**
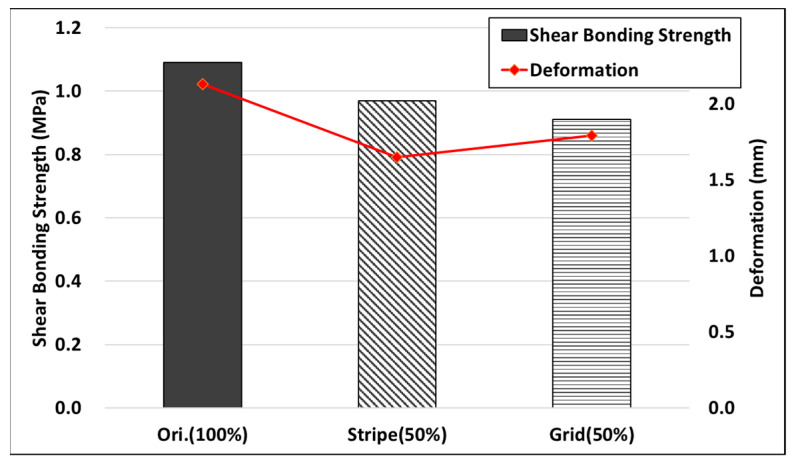
Shear bond strength due to tack coat loss.

The results of the displacement values at failure are shown in Figure 23. When there was no tack coat loss, the displacement value at failure was 2.13 mm. However, in the case of specimens with loss in stripes and lattice patterns, the displacement values were 1.65 mm and 1.79 mm, respectively. This indicates that even with small deformations, destruction occurred. Therefore, minimizing the damage of the tack coat is crucial for the durability of the pavement. The pavement can be destroyed even with a smaller deformation if it is affected by the loss caused by the construction vehicle after the spraying of the tack coat.

### 3.7. Field Test Results: Effectiveness of Conventional Tack Coating Method and Trackless Coating Method

After conducting the field test bed comparing the conventional tack coating method and the trackless coating method in the Mekong Delta region, the results provide valuable insights into the effectiveness of these approaches in improving adhesion between the old cement concrete pavement and the overlaying Warm Mix Asphalt. The performance of both sections was evaluated based on the long-term durability of the pavement after one year of service life in the challenging environmental conditions of the Mekong Delta.

**Figure 24:**
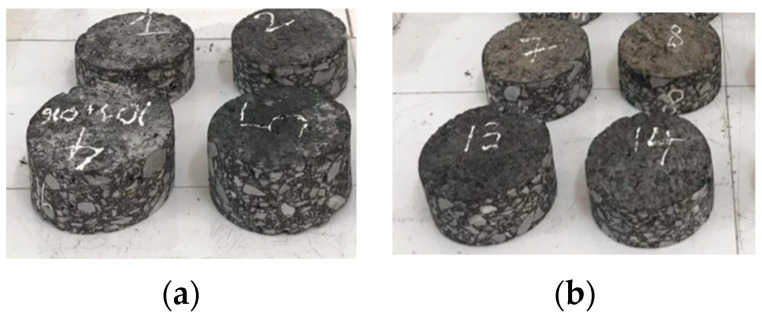
Coating comparison between the two options: (**a**) conventional method; (**b**) trackless tack-coating method.

To assess the effectiveness of the tack coating methods, various parameters were measured and compared between the control section and the modified section. The following Table 7 summarizes the findings. As observed in Figure 24 below, the newly proposed trackless tack coating exhibits a tight bonding with the aged concrete skin, indicating its excellent adhesion properties.

The field test results revealed several advantages of the trackless coating method over the conventional tack coating method. The trackless coating method exhibited superior shear bond strength, achieving a value of 1.36 MPa compared to 0.81 MPa for the conventional method, indicating enhanced adhesion between pavement layers and improved structural integrity. Additionally, the trackless coating method demonstrated a higher average compaction level of 98.5 ± 1%, surpassing the 97 ± 1% achieved by the conventional method, leading to improved pavement stability and durability. The surface condition was rated as excellent for the trackless coating method and good for the conventional method, indicating a smoother and more uniform surface, resulting in enhanced ride quality. The elastic modulus values were slightly higher for the trackless coating method (169.5 MPa) compared to the conventional method (159.5 MPa), indicating improved resistance to deformation and increased pavement stiffness. Furthermore, the trackless coating method exhibited reduced rutting with a measurement of 2.2 mm, outperforming the conventional method’s rutting measurement of 3 mm, indicating enhanced resistance to permanent deformation and rutting, ultimately contributing to improved pavement performance.

Based on the field test results, it is evident that the trackless coating method slightly outperformed the conventional tack coating method in various aspects. Consequently, the overall pavement condition in the modified section was found to be better than that of the control section. These findings demonstrate the effectiveness of the trackless coating method in improving the long-term performance of the pavement under the challenging climatic and traffic conditions of the Mekong Delta. The results highlight the importance of selecting appropriate tack-coating techniques to enhance pavement durability and maintain optimal performance over time. The data obtained from this field test bed will contribute to informed decision-making in pavement construction practices, promoting sustainable and resilient road infrastructure in similar regions.

### 3.8. Discussion

The Discussion section of this study presents the findings and analysis of various tests conducted on the newly developed tack coat material. To provide a comprehensive understanding of the effectiveness of the trackless tack coating method, the results obtained in this study will be discussed in relation to the results reported by other researchers.

In terms of the binder bond strength (BBS) test, the findings demonstrate that the newly developed tack coat material exhibits significantly higher adhesion strength compared to conventional rapid-setting products. This observation is consistent with the results reported by Wang et al. (2018) [25], who also found improved bond strength with the incorporation of Polyurethane additives in tack coat materials. Furthermore, this study highlights the influence of surface type on adhesion strength, with higher adhesive strength observed on concrete surfaces due to their quick moisture absorption capabilities. These findings align with the work of Apostolidis (2020), who emphasized the importance of moisture absorption in optimizing tack coat performance [8].

Regarding the tensile bond strength test, the results show that the new trackless tack coat outperforms the rapid-setting tack coat under all spraying conditions. This finding is in agreement with the findings of Clark et al. (2012), who investigated the impact of different tack coat materials on tensile bond strength and concluded that modified tack coats exhibit enhanced adhesion properties [15]. Furthermore, this study identified an optimal spraying amount of 0.45 l/m^2^ for achieving the highest tensile bond strength, corroborating the work of Geng et al. (2022) [24], who also reported an optimal application rate for tack coat materials.

The shear behavior analysis revealed that the trackless tack coat exhibits a significantly higher adhesion strength compared to the rapid-setting tack coat. This finding is consistent with the findings of Chen et al. (2012) [26], who investigated the shear behavior of tack coat materials and observed improved adhesion performance with modified tack coats. This study further demonstrates that the shear energy analysis indicates the potential enhancement of bond strength with the trackless tack coat, as evidenced by the higher shear reduction factor and intact adhesion up to the point of interfacial separation.

To provide a broader perspective, a comparison of the results obtained in this study with those reported by other authors supports the effectiveness of the newly developed trackless tack coat in improving adhesion strength, tensile bond strength, and shear behavior. These results are consistent with prior research by various authors, including Dian et al. (2018) [38], Seo et al. (2021) [27], and Peng et al. (2023) [25]. By considering the collective evidence, it becomes evident that the trackless tack coating method offers superior performance compared to conventional tack coating methods.

In conclusion, the discussion of the results in this study, in conjunction with the comparison to findings from other authors, supports the notion that the newly developed trackless tack coat material demonstrates improved effectiveness in terms of adhesion strength, tensile bond strength, and shear behavior. These findings contribute to the existing body of knowledge in the field and underscore the significance of the trackless tack coating method as a promising solution for enhancing pavement performance.

## 4. Conclusions

The modified emulsified asphalt was evaluated for its adhesion performance to optimize the material and ensure its field applicability. The evaluation took into consideration the characteristics of the simultaneous laying method used to improve the adhesion performance of the polymer-modified tack coat in asphalt overlay pavements of aged concrete. Based on the evaluation, the following conclusions were drawn.

1. Based on the BBS test results, the newly developed tack coat material showed significantly higher adhesion strength compared to conventional rapid-setting products, with 1.68 times higher strength on concrete and 1.78 times higher strength on asphalt. The results also reveal that the separation and removal of binder and moisture in the tack coat material greatly affect the adhesive strength, and that concrete has an advantage in quick moisture absorption, which is favorable for tack coat curing.
2. The tensile bond strength reached its maximum at an application rate of 0.45 l/m^2^ and demonstrated a tendency to decrease when the amount sprayed deviated from this value, either higher or lower.
3. The direct tensile adhesion test showed that the new polymer-modified trackless tack coat had a value 1.21 times larger than the rapid setting tack coat, demonstrating similar adhesion performance to the trackless tack coat material used in the Korean market. The experiment was followed by reattaching the separated pavement layer to evaluate the recovery of adhesive strength. The new trackless tack coat exhibited a tensile adhesive strength of 63% of the original state, indicating an advantage in ensuring pavement durability.
4. The shear adhesion test showed that the new trackless tack coat exhibited 1.90 times higher shear adhesion strength than the rapid-setting tack coat. The adhesion performance was also evaluated based on road surface texture, and the shear energy increased as a result of aggregate interlocking under the tinning simulation condition. The performance was improved by 12% compared to the cutting surface. In an experiment simulating tack coat loss by construction vehicles, the shear adhesion strength decreased when the tack coat was lost. In particular, the deformation at breakage was reduced by more than 71%, indicating that the resistance to damage decreased when the tack coat was lost. Therefore, it is believed that the laying method can be effective in preventing tack coat loss.
5. If damage occurs after spraying the tack coat, it can lower the adhesion performance and accelerate pavement damage. Therefore, the use of the new trackless tack coat appears to be an effective material for improving the adhesion performance between pavement layers without causing process delays, especially in situations where the curing time is insufficient, such as in the simultaneous laying method.
6. The field test results unequivocally confirm the significantly improved effectiveness of the trackless coating method compared to the conventional tack coating method, as demonstrated by superior shear bond strength, higher compaction levels, excellent surface condition, improved elastic modulus, and reduced rutting.
7. In general, in addition to some merits of this research, limitations of this study include the fact that the experiments were conducted at room temperature and the results may vary under different conditions. Future research could explore the effectiveness of the modified emulsified asphalt under different temperatures and weather conditions. Additionally, the long-term durability of the new trackless tack coat could be investigated through field trials.

## Figures and Tables

**Table 1:** Specification of modified emulsion asphalt.

Tests	Specification	Results	Test Method
Viscosity Saybolt Furol, sec (at 77 °F)	20–100	59	AASHTO T 72
Sieve test, %	≤0.1	0.01	AASHTO T 59
Storage stability, % (24 h)	≤1	0	
Particle charge	Positive	Positive	
Distillation test:			
Residue by distillation, % by weight	≥62	64.0	
Oil distillate, % by weight	≤0.5	-	
Tests on residue from distillation:			
Polymer content, wt, % (solids basis)	≥3.0	4.3	-
Penetration (at 77 °F, 100 g, 5 s)	55–90	68	AASHTO T 49
Solubility in trichloroethylene, %	≥97.0	99.2	AASHTO T 44
Softening Point, °C	≥135	141	AASHTO T 53
Ductility, cm (at 77 °F, 5 cm/min)	≥70	80	AASHTO T 51

**Table 2:** Results of tensile bond strength test.

Rate	0.30 l/m^2^	0.45 l/m^2^	0.60 l/m^2^
Material			
Rapid setting	0.54 MPa	0.86 MPa	0.80 MPa
New trackless	0.74 MPa	1.05 MPa	0.83 MPa

**Table 3:** Comparison of tensile bond strength with Korea-featured products.

Material	Tensile Bond Strength (MPa)	
	Origin	Re-Bonding
New Trackless	1.08	0.68
Rapid setting type A	0.99	0.06
Rapid setting type B	1.01	0.08
Rapid setting type C	0.92	0.11

**Table 4:** Result of the shear test.

Rate (l/m^2^)	Shear Bond Strength (MPa)					
	Cutting		Laitance		Tinning	
	New Trackless	Rapid Setting	New Trackless	Rapid Setting	New Trackless	Rapid Setting
0.30	0.97	0.79	1.04	0.75	1.17	1.16
0.45	1.09	0.81	1.22	0.85	1.21	1.17
0.60	0.92	0.80	1.13	0.79	1.34	1.20

**Table 5:** Analysis of Shear Test.

Item	Condition	Rate (ℓ/m^2^)	$\tau_{\max}$ (MPa)	$k_{s}$ (MPa/mm)	$k_{r}$ (MPa/mm)	Shear Energy (MPa.mm)
New trackless	Cutting	0.30	0.97	0.46	−2.31	1.24
		0.45	1.09	0.52	−2.87	1.35
		0.60	0.92	0.37	−2.24	1.32
		Avg.	0.99	0.45	−2.47	1.30
	Laitance	0.30	1.04	0.32	−2.74	1.88
		0.45	1.22	0.43	−1.97	2.11
		0.60	1.13	0.31	−2.09	2.34
		Avg.	1.13	0.35	−2.27	2.11
	Tinning	0.30	1.17	0.52	−2.85	1.55
		0.45	1.21	0.56	−3.18	1.53
		0.60	1.34	0.64	−3.94	1.62
		Avg.	1.24	0.58	−3.33	1.57
Rapid setting	Cutting	0.30	0.79	0.34	−1.72	1.11
		0.45	0.81	0.36	−1.84	1.09
		0.60	0.80	0.33	−1.78	1.15
		Avg.	0.80	0.34	−1.78	1.11
	Laitance	0.30	0.75	0.34	−1.21	1.05
		0.45	0.85	0.30	−1.73	1.42
		0.60	0.79	0.41	−1.52	0.97
		Avg.	0.80	0.35	−1.49	1.15
	Tinning	0.30	1.16	0.51	−2.27	1.62
		0.45	1.17	0.52	−2.39	1.60
		0.60	1.20	0.55	−2.67	1.58
		Avg.	1.18	0.53	−2.44	1.60

**Table 6:** Shear bond strength due to tack coat loss.

Condition	Shear Bonding Strength (MPa)	Deformation (mm)
Original (100% tack coat covering)	1.09	2.13
Stripe (50% tack coat covering)	0.97	1.65
Grid (50% tack coat covering)	0.91	1.79

**Table 7:** General field test results.

Parameter	Conventional Tack Coating Section	Trackless Tack Coating Section
Shear Bond Strength (MPa)	0.81	1.36
Average Compaction Level	97 ± 1%	98.5 ± 1%
Overall Surface Condition	Good	Excellent
Elastic Modulus (MPa)	159.5	169.5
Rutting on Section	3±0.5 mm	2.2 ±0.5 mm

## Data Availability

Data will be made available on request.
